# Adult residual rectourethral fistula and diverticulum presenting decades after imperforate anus repair: a case report

**DOI:** 10.1186/s13256-021-02921-3

**Published:** 2021-07-15

**Authors:** Erin K. McShane, Brooke Gurland, Vipul R. Sheth, Matias Bruzoni, Ekene Enemchukwu

**Affiliations:** 1 Stanford University School of Medicine, 291 Campus Drive, Stanford, CA 94305 USA; 2 Stanford University Medical Center, 300 Pasteur Drive, Stanford, CA 94305 USA

**Keywords:** Case report, Imperforate anus, Rectourethral fistula, Posterior sagittal anorectoplasty, Robotic procedure

## Abstract

**Background:**

This report describes a rare surgical case of an intraabdominal mass in a middle-aged patient 40 years after imperforate anus repair.

**Case presentation:**

A 44-year-old Latino male with history of repaired anorectal malformation presented with recurrent urinary tract infections and rectal prolapse with bothersome bleeding and fecal incontinence. During his preoperative evaluation, he was initially diagnosed with a prostatic utricle cyst on the basis of magnetic resonance imaging findings, which demonstrated a cystic, thick-walled mass with low signal contents that extended inferiorly to insert into the distal prostatic urethra. However, at the time of surgical resection, the thick-walled structure contained an old, firm fecaloma. The final pathology report described findings consistent with colonic tissue, suggesting a retained remnant of the original fistula and diverticulum.

**Conclusions:**

Although rare, persistent rectourethral fistula tracts and rectal diverticula after imperforate anus repair can cause symptoms decades later, requiring surgical intervention. This is an important diagnostic consideration for any adult patient with history of imperforate anus.

**Supplementary Information:**

The online version contains supplementary material available at https://doi.org/10.1186/s13256-021-02921-3.

## Introduction

**Fig. 1:**
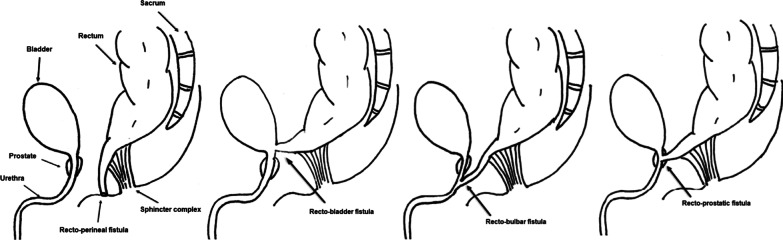
Variants of anorectal malformation by M. Bruzoni

Anorectal malformation (ARM), also known as imperforate anus, is one of the most commonly observed congenital defects affecting between 1/2500 and 1/5000 live births [1, 2]. In ARM, the anal opening is either absent or displaced so there is no external opening from the rectum. The different types of ARM are defined by the location of the end of the bowel in relation to the pelvic floor and the genitourinary system. In males, the three commonly described ARMs include rectoperineal fistula, rectourethral fistula (prostatic or bulbar fistula), and rectobladder fistula (Fig. 1).

Posterior sagittal anorectoplasty (PSARP), introduced in 1982, has been the standard-of-care treatment for ARM. Laparoscopic division of the fistula and coloanal pull-through has been popularized in the last 15 years [1, 3]. In all procedures, the fistula is divided, but a remnant diverticulum can remain if excision of the fistula was overly conservative in avoiding urethral damage [4]. Over time, this tract can enlarge and epithelialize to form a diverticular structure arising from the posterior urethra [4]. In prior reports, patients presenting with retained remnant of the original fistula (ROOF), previously called posterior urethral diverticulum, were younger (mean 4.5 years) with a history of recurrent urinary tract infections (UTIs) and a perineal mass palpable on rectal examination [5]. This report reviews a case of diverticulized residual fistula tract in an adult patient decades after ARM repair.

## Case presentation

A 44-year-old Latino male with a history of anorectal malformation presented to colorectal surgery clinic with chronic, progressive rectal pain exacerbated by walking and other symptoms of rectal prolapse including rectal bleeding, fecal urgency, and incontinence. As an infant, he underwent colostomy with subsequent imperforate anus repair. However, postoperative reports and surgical details were not available for review.

He reported a 30-year history of intermittent dysuria, pneumaturia, urinary frequency, recurrent UTIs, and fecaluria. He explained that from the age of 12 or 14 years, each time he had diarrhea, he would subsequently develop a UTI. With age, the infections grew more frequent and debilitating. He regularly missed school or work because of pain. In his 20s, he developed rectal prolapse that led to bothersome episodes of bleeding—especially while performing heavy lifting at work. He began using sanitary napkins to protect his clothing, and transitioned to adult diapers at age 28, which he reported was accompanied by feelings of shame. He had trouble standing for more than 1 hour owing to pelvic floor exhaustion. He did not have access to health insurance and therefore did not seek help for these symptoms. He was ultimately referred to specialty care because of his recurrent UTIs and severe rectal prolapse.

## Clinical findings

On physical examination, the patient had 2–3 cm full-thickness rectal prolapse, which was largest on the right side with a patulous anus. The abdomen was soft, nontender, and nondistended with no palpable masses. In addition, he had a widened hiatus and leftward displaced anus due to prolapsing perirectal fat and sacrococcygeal dysgenesis.

### Timeline

Figure 2.

**Fig. 2:**
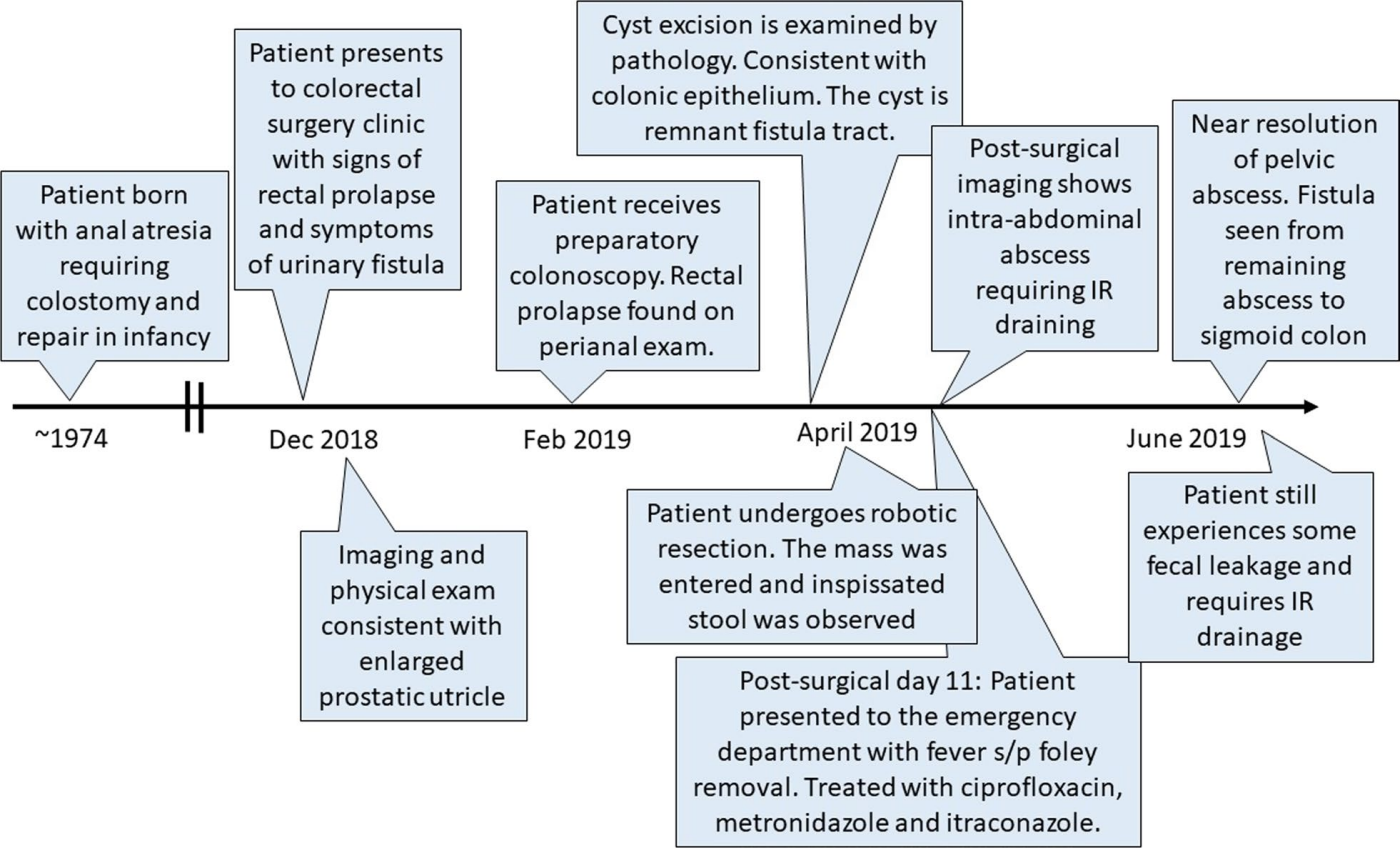
Timeline of patient’s course

### Diagnostic assessment

**Fig. 3:**
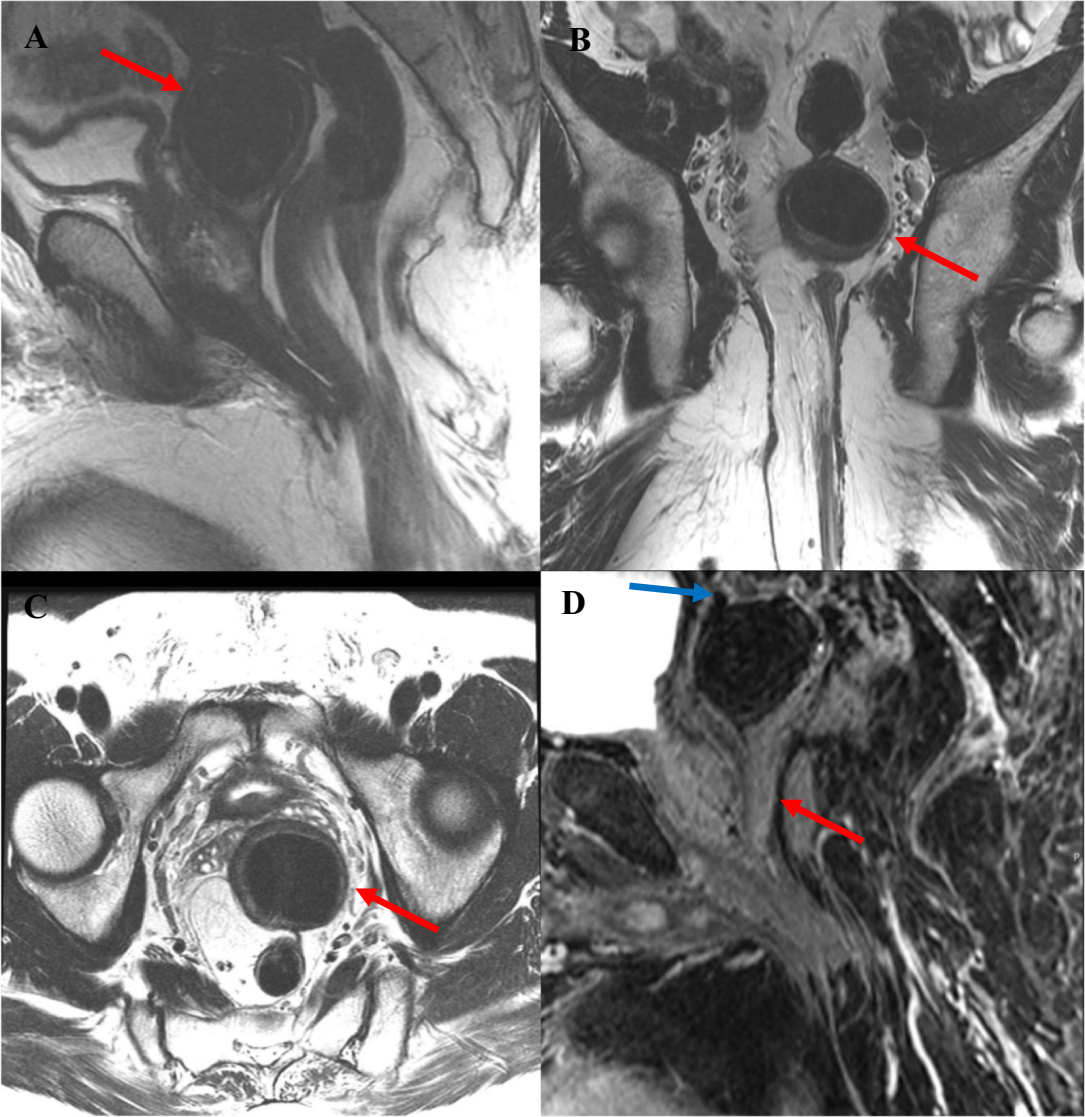
**A** Preoperative sagittal T2-weighted image showing large thick-walled T2 hypointense structure posterior and superior to the prostate (red arrow) with possible fistula to the sigmoid colon at its superior aspect. There is also a fibrous band or adhesion between the proximal rectum and distal rectum or anal canal. **B** Coronal image again showing thick-walled mass with adhesion to the distal sigmoid colon (red arrow). This image also demonstrates prolapse or perirectal fat and rectum through the external sphincter. **C** Axial image showing relationship of rectum, unknown mass (red arrow), seminal vesicles, and bladder. **D** Sagittal oblique postcontrast images again showing apparent fistula to sigmoid colon (blue arrow) and enhancing tract extending toward the distal prostatic urethra (red arrow)

Diagnostic magnetic resonance imaging (MRI) findings demonstrated a thick-walled cystic lesion with fistulous tracts extending from the superior and posterior aspect of the mass to the distal colon (Fig. 3). In a multidisciplinary conference of colorectal surgery, urology, and radiology, the presumptive diagnosis of a prostatic utricle with two possible fistulas to the prostate and colon was made. However, colonoscopy did not reveal a fistulous connection and was otherwise normal. Cystoscopy identified a high bladder neck, with no definitive connection between the prostatic, membranous, or distal prostatic urethra and the cystic mass. Urinalysis was positive for blood (4–5 red blood cells/high-powered field) and leukocyte esterase with 21–50 white blood cells/high-powered field. The final urine culture was negative for bacterial growth. The patient elected for surgical excision of the abdominal mass at the time of rectal prolapse repair.

### Therapeutic intervention

The patient underwent robotic excision of pelvic mass and a perineal proctectomy to correct his rectal prolapse (Additional file 1). Intraabdominally, the 7 × 5 × 4 cm cystic mass was carefully dissected from surrounding soft tissue. To avoid injuring the external urinary sphincter muscle, we did not dissect and excise the distal portion of the sinus tract. Therefore, we opened the cystic mass, observing firm yellow-brown contents consistent with old stool. We excised the cyst wall and copiously irrigated the pelvis with saline. The sinus tract remnant was closed in two layers with polyglactin suture, the urethra retrofilled with saline, and the repair was watertight. We secured peritoneum over the repair to provide a layer of interposition tissue. Pathological evaluation of the cystic mass described colonic tissue.

### Follow-up and outcomes

**Fig. 4:**
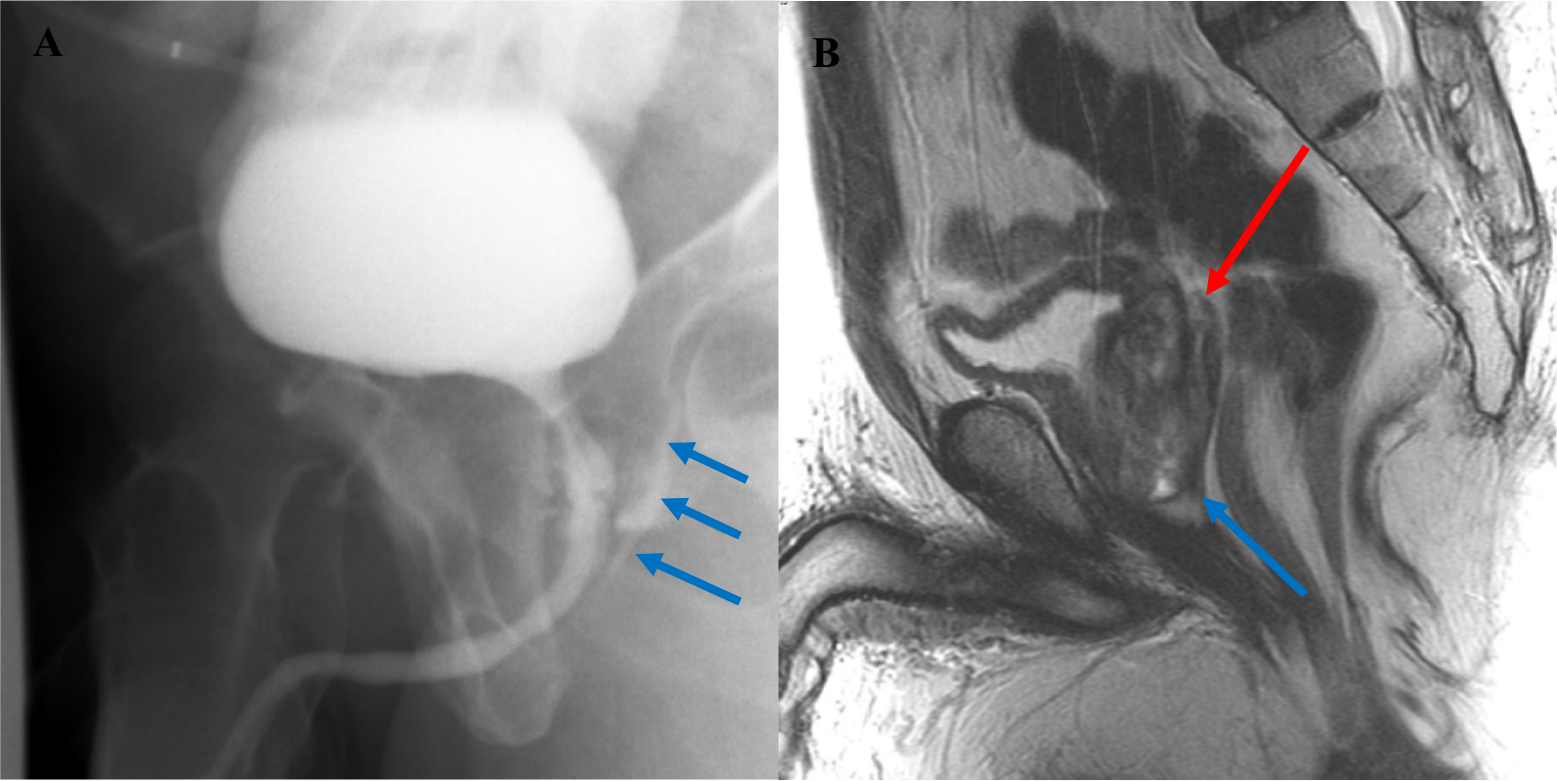
**A** Postoperative voiding cystourethrogram shows small sinus tract posterior to urethra (arrows), but no fistula to rectum. **B** Postoperative T2-weighted MRI shows resection of the cystic structure. Linear structure posterior to prostate corresponds to sinus tract on cystourethrogram (red arrow at superior border, blue arrow at most distal border). No residual abscess was seen

There were no intraoperative complications. Despite administration of oral cephalexin (250 mg daily) for UTI prevention, the course was complicated by fever on postoperative day 3 (38.9 °C) for which his antibiotics were broadened to intravenous piperacillin–tazobactam. Blood and urine cultures showed no growth at that time. He also developed a bowel ileus requiring nasogastric tube decompression. He was discharged on postoperative day 6 with an indwelling Foley catheter and returned to urology clinic 10 days after surgery for a voiding cystourethrogram (Fig. 4a), which demonstrated a sinus tract remnant in the expected location of the distal prostatic urethra but no rectal fistula. Postoperative MRI shows complete resection of the abdominal mass with no remaining abscess (Fig. 4b). The indwelling Foley catheter was removed, and instructions were given to complete his postoperative course of antibiotics.

He presented on postoperative day 11 with fever of 39.4°C and a pelvic abscess. He was admitted, and fever resolved after initiation of intravenous ceftriaxone. On hospital day 2, he was treated with a pelvic drain and began intravenous vancomycin (discontinued hospital day 3) and piperacillin–tazobactam (discontinued hospital day 9). Abscess fluid culture was positive for *Bacteroides fragilis*, *Parabacteroides distasonis*, *Candida glabrata*, *Escherichia coli*, *Lactobacillus* species, and *Enterococcus* species, and caspofungin (70 mg, then 50 mg intravenous) was added to his antibiotic regimen. He was discharged on hospital day 9 on oral ciprofloxacin, metronidazole, and itraconazole. Resolution of the pelvic abscess was noted 28 weeks postoperatively, and his drain was removed. At 1-month follow-up, the patient reported resolution of his symptoms and marked improvement in his quality of life.

By phone interview 24 months after surgery, he reported his “life completely changed” from the intervention. He reports no UTIs or bleeding, though he continues to wear adult diapers as a precaution to avoid accidents from incontinence. His surgical care also had a great positive impact on his family. He expressed he can better support his family and spends more quality time with his children. He also added excitedly, “I can stand for eight hours, no problem,” which is helpful for his work. He would “definitely” recommend this surgery to anyone with rectal prolapse, and he was happy to share his story in the hopes it helps other patients.

## Discussion

Surgical repair of anorectal malformations is associated with a number of complications, including diverticulized remnant fistula tracts and large utricular pouches just above the fistula [6]. For surgical repair in males, dividing the fistula close to the urethra is recommended to avoid a remnant diverticulum [5]. The prostatic utricle is a normal anatomic finding, but enlarged prostatic utricles can be pathologic and are commonly associated with imperforate anus [4, 6]. The anatomic locations of prostatic utricle cysts and ROOFs are virtually indistinguishable. A definitive diagnosis may be made if preoperative imaging is available from imperforate anus repair. Otherwise, pathology is necessary [4]. We first considered a diagnosis of ROOF during surgery after observing inspissated stool in the excised mass. The patient’s constellation of symptoms was most consistent with ROOF, including fecaluria and recurrent UTIs. Given the presence of colonic tissue, the final pathology report was most consistent with a diagnosis of ROOF.

Although imperforate anus is a common congenital anomaly, it is rare to see complications from the anomaly or repair manifest in adulthood. To our knowledge, this case is a unique presentation of an ARM repair complication in adulthood. Odaka *et al*. reported a case of a 48-year-old man with rectourethral fistula and a bladder diverticulum; however, he never underwent ARM repair, instead undergoing permanent colostomy [7]. Toyama reported the case of a 28-year-old man with a high rectourethral fistula who also experienced recurrent urinary tract infections [8]. Similarly, this patient had only been treated with colostomy and had no correction of their congenital rectourethral fistula. Neither of these patients developed a stool-filled diverticulum presented in this report. Nakayama *et al*. reported the case of a man with a history of imperforate anus and sigmoid loop colostomy, which was converted to left lower abdomen sigmoid divided colostomy at age 28 years [9]. He later presented at age 71 years with a rectourethral fistula and megarectum, which was subsequently repaired.

More often, ROOF has been reported in pediatric patients (~2% of repairs) [5]. Rentea *et al*. provide a retrospective cohort study to better understand the occurrence and presentation. Of 180 male patients referred for urinary and/or fecal continence concerns after surgical repair, 16 had ROOF. Fourteen of these patients required surgical repair, and 13 had a secondary indication for surgery (rectal prolapse or anal mislocation). The mean age for these patients was 4.5 years (range 2–7 years). A larger study by Alam *et al*. observed a cohort of 260 male patients presenting with complications after ARM repair [10]. In this cohort, there was a greater range in age at presentation, with the latest presentation at 24 years after surgery (median 9 years). Of note, they described the typical presentation of ROOF/posterior urethral diverticulum in which the diverticulum develops from a retained part of the rectourethral fistula and enlarges as urine is retained in the pouch-like structure. This exposure of colonic mucosa to urine is known to increase risk for adenocarcinoma [10]. In contrast, our patient retained fecal matter rather than urine. Upon surgical resection, the mass contained dried stool. His occasional fecaluria suggests that he similarly had mixing of urine and fecal matter. However, he has no history of adenocarcinoma, and it is unclear whether his presentation carries the same risk as patients with a typical presentation of ROOF.

There are a variety of factors that could explain our patient’s late presentation of complications from anorectal malformation repair. First, he may have experienced milder symptoms than most other patients. He reported his first complications around age 12 years, which is beyond the median age from both studies we previously discussed. The fecal versus urine retention may have contributed to this progressive onset of symptoms. Additionally, access to care greatly contributed to his delayed presentation. With proper follow-up care, he might have received surgical correction promptly after onset of his symptoms. We anticipate that other patients without follow-up care might suffer complications from imperforate anus repair into adulthood with delayed reporting. Enhanced awareness of this postsurgical sequela among pediatric surgeons would facilitate better patient education, while empowering patients to seek early follow-up care with any symptoms of rectourethral fistula. Furthermore, better awareness among primary care physicians and colorectal surgeons could facilitate earlier surgical intervention in conjunction with improved access to care.

## Conclusions

We present the case of a 44-year-old male with symptoms of a retained remnant of the original fistula (ROOF) years after repair of anorectal malformation. Our patient experienced recurrent UTIs in his adolescence and rectal prolapse in his 20s but faced several barriers to seeking care, including lack of healthcare coverage. Proper diagnosis of urinary and colorectal complications in adult patients with history of ARM is complex and is best addressed with a multidisciplinary team including primary care, urology, colorectal surgery, pediatric surgery, and radiology. Early evaluation and treatment in patients with symptoms of ROOF could improve patient quality of life following ARM repair complications.

## Supplementary Information

**Additional file 1.** Video case report with footage of and commentary on robotic excision and perineal proctectomy.

## Data Availability

Data sharing is not applicable to this article as no datasets were generated or analyzed during the current study.

## Abbreviations

ARM: Anorectal malformation
ROOF: Retained remnant of the original fistula
UTI: Urinary tract infection
MRI: Magnetic resonance imaging
PSARP: Posterior sagittal anorectoplasty
